# Predictive factors for HbA1c and weight loss associated with semaglutide treatment in type 2 diabetes mellitus: real-world clinical evidence

**DOI:** 10.3389/fendo.2025.1621892

**Published:** 2025-09-16

**Authors:** Petya Milushewa, Yoanna Mitreva, Nevena Chakarova, Tsvetalina Tankova, Emilia Naseva, Valentina Petkova

**Affiliations:** ^1^ Department of Organization and Economics of Pharmacy, Medical University of Sofia, Sofia, Bulgaria; ^2^ Department of Endocrinology, Medical University of Sofia, Sofia, Bulgaria; ^3^ Department of Health Management and Health Economics, Medical University of Sofia, Sofia, Bulgaria

**Keywords:** semaglutide, type 2 diabetes, obesity, glycemic control, weight loss, real-world data

## Abstract

**Objective:**

This study aimed to identify clinical predictors associated with reductions in HbA1c and weight among patients with type 2 diabetes mellitus (T2DM) treated with semaglutide in real-world clinical settings in Bulgaria.

**Methods:**

A retrospective analysis was conducted on 168 T2DM patients receiving dispensary monitoring at the University Specialized Hospital for Active Treatment in Endocrinology “Acad. Ivan Penchev,” Sofia. Clinical data, including comorbidities, HbA1c, weight, and BMI, were collected from medical records at baseline and after one year of treatment. Patients were administered subcutaneous semaglutide beginning at 0.25 mg weekly, titrated to 0.5 mg after four weeks, and to 1 mg thereafter, maintained over a one-year period.

**Results:**

At baseline, 92.3% of patients had obesity (BMI ≥30 kg/m²), with 53.6% categorized as Obesity Class 1. After one year of GLP - 1RA therapy, significant improvements in metabolic parameters were observed. Median weight reduced from 100.0 kg to 91.5 kg (p<0.001), and median BMI decreased from 33.6 to 30.9 kg/m² (p<0.001). HbA1c levels declined from 7.80% to 6.90% (p<0.001). Transitions to lower obesity classes occurred in 81 patients, while 15 remained in Class 3 obesity.

**Conclusions:**

Semaglutide significantly improves glycemic control and promotes weight and BMI reduction in T2DM patients in routine clinical practice. These findings suggest that structured follow-up under endocrinologist supervision, as required by national protocols, may enhance therapeutic response. Further prospective studies with extended follow-up are warranted to evaluate the long-term sustainability of these effects and to identify predictors of optimal patient response.

## 1 Introduction

Obesity is defined by (1) the World Health Organization as an “abnormal or excessive fat accumulation that poses a risk to health” and is widely recognized as a pressing global health issue (1). In the European Union, more than half of the adult population aged 35 years and older is classified as overweight, with similar patterns observed in Bulgaria (2). Obesity is a recognized concern within Bulgaria’s pediatric population, with prevention identified as a priority in the National Strategy for Children’s and Adolescents’ Health and Pediatric Care. Despite the acknowledgment of obesity as a public health priority in national strategies, Bulgaria lacks a dedicated program addressing obesity specifically in adults.

Obesity is closely associated with type 2 diabetes mellitus (T2DM), with over 85% of individuals diagnosed with T2DM also exhibiting overweight or obesity (3). The overlap in pathophysiology between these conditions - often referred to as “diabesity” - is underpinned by shared mechanisms such as insulin resistance, disrupted hormonal signaling, and altered energy metabolism (4, 5). Recent data indicate a marked rise in the prevalence of diabetes in Bulgaria, increasing by over 7% between 2019 and 2021 (6). This underscores the urgency of implementing effective therapeutic approaches targeting both glycemic control and weight management. Among the pharmacological options available, GLP - 1 RAs have demonstrated considerable efficacy in managing both T2DM and obesity (5). Semaglutide, a once-weekly injectable GLP - 1 RA, has been shown to significantly reduce HbA1c levels and body weight in clinical and real-world settings (7). Recent studies emphasize the role of baseline BMI in modulating treatment response to semaglutide, with individuals presenting higher initial BMI often achieving greater reductions in body weight and HbA1c. This observation is supported by clinical evidence showing that semaglutide induces preferential reductions in visceral adiposity and fat mass, which are closely associated with higher BMI categories (8). Additionally, BMI serves as a key eligibility criterion for semaglutide reimbursement and initiation in many healthcare systems, including Bulgaria, underscoring its dual role as both a clinical and regulatory determinant of therapy (9).

While these effects are well established, interindividual variability in treatment response remains insufficiently studied, particularly within real-world cohorts from Eastern Europe. This study aims to identify clinical predictors - specifically baseline BMI - that may influence the degree of HbA1c reduction and weight loss in individuals with T2DM receiving subcutaneous semaglutide in routine clinical practice. We hypothesize that patients with higher baseline BMI will exhibit more pronounced improvements in glycemic control and weight outcomes over a one-year treatment period.

## 2 Materials and methods

### 2.1 Study design and patient recruitment

This retrospective study comprises 168 patients with type 2 diabetes mellitus (T2DM) who undergo dispensary monitoring at the Department of Diabetology of the University Specialized Hospital for Active Treatment in Endocrinology “Acad. Ivan Penchev,” Sofia. According to the established clinical practice in Bulgaria dispensary monitoring refers to mandatory, structured follow-up evaluations conducted at 6-month intervals, as required by the NHIF, for the continued prescription and reimbursement of high-cost medications such as semaglutide. These follow-up assessments serve to verify therapeutic efficacy - specifically, a reduction in HbA1c of at least 1% and a weight loss of at least 3% at the 6-month mark, as well as the maintenance of clinical benefit after 12 months. Adherence to treatment is confirmed through documented prescription dispensing, which is a prerequisite for protocol renewal and continued reimbursement authorization under NHIF regulations. The extracted data includes medical records of dispensary patients with both baseline and follow-up information available within a one-year period. The collected data encompasses general clinical status, T2DM-related complications, HbA1c test results, weight, and BMI. The patients received semaglutide therapy according to the following regimen: 168 patients administered subcutaneous semaglutide at an initial dose of 0.25 mg weekly for 4 weeks, gradually titrated to 0.5 mg weekly for 4 weeks and thereafter 1 mg weekly. The period of analysis was 1 year.

### 2.2 Inclusion and exclusion criteria

All patients with a confirmed diagnosis of type 2 diabetes mellitus (T2DM) undergoing structured dispensary monitoring at the Department of Diabetology, University Specialized Hospital for Active Treatment in Endocrinology “Acad. Ivan Penchev,” Sofia, were screened for eligibility. Inclusion required fulfillment of the National Health Insurance Fund (NHIF) criteria for 100% reimbursement of GLP - 1 receptor agonist (GLP - 1RA) therapy with semaglutide. Specifically, patients were eligible if they had a body mass index (BMI) ≥30 kg/m² and an HbA1c level >7.5% despite at least three months of oral glucose-lowering therapy. There were no additional exclusion criteria beyond these clinical thresholds. Patients were excluded if they were not prescribed semaglutide, were hospitalized during the observation period, or lacked a confirmed diagnosis of T2DM. To ensure data completeness and integrity, only individuals with full baseline and 12-month follow-up data for key parameters (HbA1c, body weight, and BMI) were included. All measurements were performed under standardized conditions, including fasting state assessments and the use of the same calibrated equipment at each visit. Additionally, all participants received uniform dietary and physical activity counseling, in accordance with the institution’s diabetes care protocol. The accuracy of the retrospective data was ensured through systematic double-checking of hospital medical records.

### 2.3 Institutional review board statement

This study was conducted in accordance with national legal requirements and received approval from the Medical University-Sofia Research Ethics Commission (Protocol Nr 02/20.02.2025).

### 2.4 Statistical analysis

The results are presented as numbers or proportions of the patients for categorical variables, and median and interquartile range (IQR) for numerical due to their non-Gaussian distribution. However, mean values are reported for comparability to other studies. The distribution was assessed by Kolmogorov-Smirnov test. Wilcoxon Singed Rank test was used to assess the change between two time points (baseline and 12th month). Binary logistic regression was used to test factors for HbA1c improvement and weight loss. The results were considered significant if p<0.05. The results were analyzed by using IBM SPSS v. 26.

## 3 Results

### 3.1 Demographic characteristics

The median age of the participants was 57 years old (IQR 49 - 62.8), ranging from 20 to 77 years old. Most participants (n=93) were aged 40–59 years, representing over half of the study population (55.4%). In contrast, the youngest age group (20–39 years) was the smallest, comprising only 14 participants (8.3%) of the sample. The gender distribution was nearly balanced, with 90 female patients (53.6%) and 78 male patients (46.4%). Regarding disease duration, a significant portion of the cohort (n=36) had been diagnosed with T2DM for less than 1 year, indicating a notable inclusion of newly diagnosed cases. Patients with a disease duration exceeding 5 years accounted for 44% of the cohort (**Table 1**).

**Table 1:** Demographic characteristics of patients.

Characteristic	n	%
Age (yrs)		
20-39	14	8.3
40-59	93	55.4
60-79	61	36.3
Gender		
Male	78	46.4
Female	90	53.6
Duration of T2DM (yrs)		
Below 1	36	21.2
1-2	34	20.2
3-5	23	13.7
6-10	28	16.7
11-20	39	23.2
Above 20	8	4.8

### 3.2 Baseline BMI distribution

The distribution of patients across BMI categories at the initiation of semaglutide therapy is summarized in **Table 2**. Notably, most of the patients (92.3%) had a BMI within the obesity range (≥30 kg/m²) at the start of treatment. Among these, the largest proportion (n=90, 53.6%) were classified as having obesity class 1, while a smaller subset (n=13, 7.7%) were categorized as overweight (BMI 25–29.9 kg/m²).

**Table 2:** BMI categories at the initiation of semaglutide therapy.

BMI, kg/m^2^	N	%
25 to 29.9: Overweight	13	7.7
30 to 34.9: Obesity class 1	90	53.6
35 to 39.9: Obesity class 2	41	24.4
≥ 40: Obesity class 3	24	14.3

### 3.3 Diabetes-related complications

The prevalence of T2DM-related complications among the study population is presented in **Table 3**. Diabetic neuropathy was the most common complication, affecting 63 patients (37.5%). Diabetic macroangiopathy, indicative of vascular complications such as peripheral arterial disease or coronary artery disease, was reported in 29 patients (17.6%).

**Table 3:** T2DM-related complications.

Type of complication	N	%
Diabetic neuropathy	63	37.5
Diabetic nephropathy	14	10.3
Diabetic retinopathy	12	7.1
Diabetic macroangiopathy	29	17.3

### 3.4 Changes in metabolic parameters after one year

**Figure 1:**
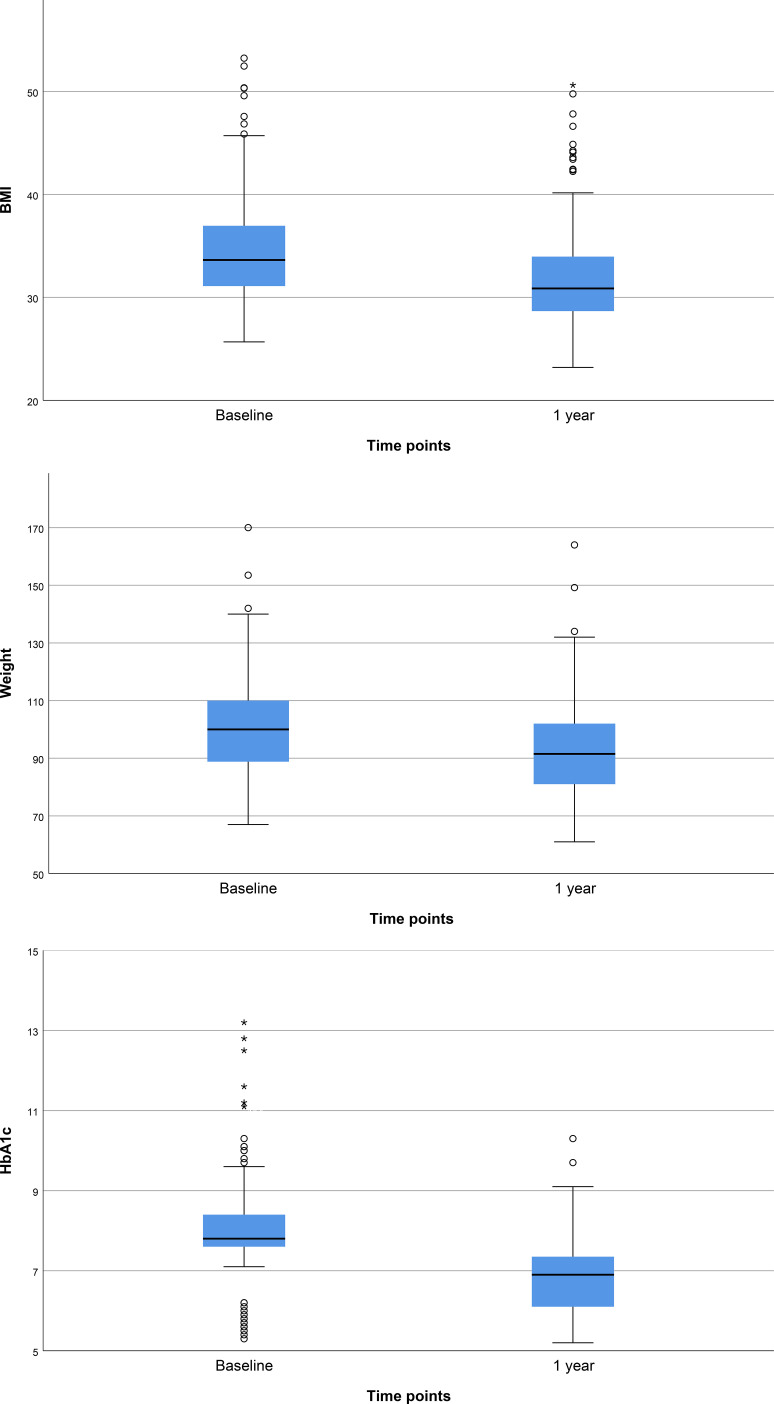
Baseline and 1-year follow-up metabolic parameters: BMI; Weight; HbA1c.

**Figure 1** presents the baseline and 1-year follow-up characteristics of the study cohort. The median baseline weight was 100.0 kg, IQR 88.8 - 110.0 (mean 101.7 kg), which decreased significantly to 91.5 kg (mean 93.6 kg), IQR 81.0 - 102.0, (p<0.001) after 1 year of treatment. Similarly, the baseline median BMI was 33.6 kg/m², IQR 31.0 - 37.0 (mean 34.8), indicative of obesity (BMI ≥30 kg/m²). Following 1 year of GLP - 1RA therapy, the BMI decreased to median 30.9 kg/m² (mean 32.0), IQR 28.7 - 34.0 (p<0.001) nearing the lower threshold of obesity class 1. Glycemic control also improved notably during the follow-up period. The median baseline HbA1c was 7.80% (mean 8.05), IQR 7.60 - 8.40, indicating suboptimal control at the start of therapy. After 1 year of treatment, HbA1c levels improved significantly to 6.90% (mean 6.89), IQR 6.10 - 7.35 (p<0.001), approaching recommended targets for patients with T2DM.

### 3.5 Subgroup analysis by BMI class

**Figure 2:**
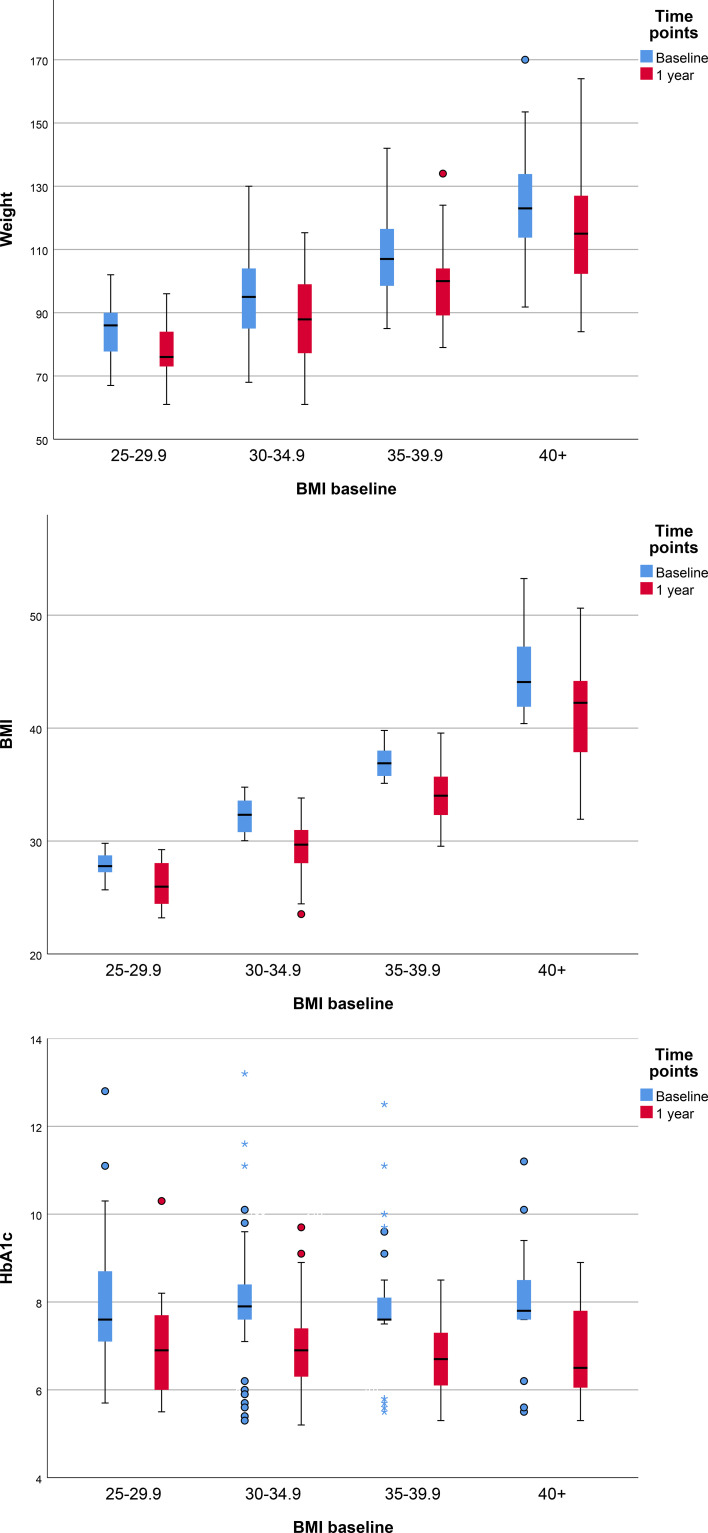
Changes in Weight, BMI, and HbA1c after 1 year of treatment in overweight and obese groups.

We analyzed the changes in weight, BMI, and HbA1c after one year of treatment with semaglutide, stratified by baseline BMI categories: overweight, Obesity Class 1, Obesity Class 2, and Obesity Class 3 (**Table 4**, **Figure 2**). All groups demonstrated statistically significant improvements in these parameters over the 12-month period (p < 0.05). Median and mean values for weight, BMI, and HbA1c declined across all subgroups, indicating consistent treatment efficacy regardless of initial BMI classification.

**Table 4:** Change in BMI categories from baseline to 12th month.

BMI at baseline	25-29.9		30-34.9		35-39.9		40+		Total
	n	% from baseline	n	% from baseline	n	% from baseline	n	% from baseline	n
25-29.9	13	100.0%	0	0.0%	0	0.0%	0	0.0%	13
30-34.9	51	56.7%	39	43.3%	0	0.0%	0	0.0%	90
35-39.9	1	2.5%	24	60.0%	15	37.5%	0	0.0%	40
40+	0	0.0%	2	8.7%	6	26.1%	15	65.2%	23
Total	65	39.2%	65	39.2%	21	12.7%	15	9.0%	166

### 3.6 Transitions in BMI classification

**Table 5** presents the distribution of BMI category changes among patients after one year of follow-up; however, the analysis was not stratified by sex or age, and potential variations across these demographic subgroups were not explored. A substantial number of patients demonstrated clinically meaningful reductions in BMI. Notably, 51 individuals transitioned from Obesity Class 1 to the overweight category, while 39 remained in the same class. Among those initially classified as Obesity Class 2, 24 moved to Obesity Class 1, and one patient reached the overweight range. For patients with severe obesity (Obesity Class 3), six transitioned to Obesity Class 2 and two to Class 1, while 15 remained in the highest BMI category. Overall, the distribution demonstrates a consistent downward shift in BMI categories over the 12-month period, with no participants moving into higher BMI classifications. This pattern reflects a favorable treatment effect across all baseline BMI groups. BMI transition data were unavailable for two patients. 

**Table 5:** Change in the BMI class at the first year.

Groups	Measure	Baseline				1 year				Difference baseline-1 year		P
		Median	Percentile 25	Percentile 75	Mean	Median	Percentile 25	Percentile 75	Mean	Median	Mean	
25-29.9	Weight	86.0	77.7	90.0	83.9	76.0	73.0	84.0	77.9	2.3	1.9	0.004
	BMI	27.8	27.2	28.7	28.0	26.0	24.4	28.1	26.1	6.0	5.9	0.005
	HbA1c	7.60	7.10	8.70	8.29	6.90	6.00	7.70	7.05	1.0	1.2	0.002
30-34.9	Weight	95.0	85.0	104.0	95.6	87.9	77.2	99.0	87.8	2.3	2.6	<0.001
	BMI	32.3	30.8	33.6	32.2	29.7	28.0	31.0	29.6	7.0	7.8	<0.001
	HbA1c	7.90	7.60	8.40	8.07	6.90	6.30	7.40	6.94	1.0	1.1	<0.001
35-39.9	Weight	107.0	98.5	116.5	107.8	100.0	89.2	104.0	99.1	2.5	3.0	<0.001
	BMI	36.9	35.8	38.0	37.1	34.0	32.3	35.7	34.1	7.5	8.7	<0.001
	HbA1c	7.60	7.60	8.10	7.94	6.70	6.10	7.30	6.76	1.1	1.2	<0.001
40+	Weight	123.0	110.0	135.0	124.8	115.0	102.0	130.0	115.4	2.8	3.4	<0.001
	BMI	44.1	41.9	47.6	45.0	42.2	37.7	44.3	41.5	7.4	9.4	<0.001
	HbA1c	7.80	7.60	8.50	7.99	6.50	6.05	7.80	6.82	1.5	1.2	<0.001

### 3.7 Factors for HbA1c improvement and weight loss

Improvement was defined as a reduction in all three parameters: HbA1c, weight, and BMI, compared to baseline. Twenty-four patients (14.5%) did not meet this criterion for improvement: one participant showed no improvement in any parameter, five showed improvement in only one parameter, and the remaining eighteen improved in two parameters. Univariate analysis identified only one significant factor: baseline HbA1c level. Specifically, each 1% increase in baseline HbA1c was associated with a 152% higher likelihood of achieving improvement in 12 months (**Table 6**). This analysis was adjusted for age, sex, and disease duration.

**Table 6:** Univariate logistic regression for predictors of treatment response.

Factor	P	OR	OR 95% CI	
HbA1с%	0.047	1.525	1.005	2.313

## 4 Discussion

Our findings confirm that reductions in HbA1c, as well as absolute and relative weight reductions achieved with injectable semaglutide, are clearly observed in real-world clinical practice. The validity of our findings is reinforced by recent studies evaluating the effectiveness of semaglutide in promoting weight loss in both patients with T2DM who followed the standard titration regimen, starting at 0.25 mg once weekly. Notably, our results align with those of a recent UK study, which reported a weight reduction from 110.4 ± 24.6 kg at baseline to 99.9 ± 24.9 kg after 12 months of semaglutide therapy, further supporting the efficacy of semaglutide in weight management. Comparable reductions in BMI were observed in both studies, further affirming the efficacy of semaglutide in weight management. Likewise, glycemic outcomes in our study and the UK study demonstrated significant improvements, emphasizing the consistent effectiveness of semaglutide in achieving glycemic control across diverse populations and clinical settings (10). A study conducted in Italy analyzing the real-world effects of semaglutide on glycemic control and weight management similarly demonstrated results consistent with our findings. After 12 months, the study reported a mean weight reduction of −9.3 ± 7.5 kg and a BMI reduction of −3.4 ± 2.6 kg/m² in the injectable semaglutide group, aligning with the weight and BMI reductions observed in our cohort. Furthermore, the study highlighted superior weight and BMI reductions in injectable semaglutide group compared to the oral semaglutide group over extended treatment periods, indicating the greater effectiveness of injectable semaglutide in achieving sustained weight loss (11). Our findings are consistent also with a recent study from Greece that assessed the effectiveness of semaglutide in obese individuals without T2DM. In that study, a median weight reduction of 7.4 kg (6.6% of baseline weight) was reported after three months of semaglutide therapy. Furthermore, the baseline BMI of 39.7 kg/m² in the Greek cohort decreased significantly, with reductions of 13.6% and 12.8% observed at maintenance doses of 1 mg and 2 mg, respectively (12). In our study, a median weight reduction of 8.5 kg (from 100.0 kg to 91.5 kg) was observed after one year of GLP - 1RA therapy, with the mean weight decreasing from 101.7 kg to 93.6 kg (p<0.001). This clinically significant reduction reflects some variability in individual response, potentially influenced by factors such as treatment duration, adherence, baseline BMI, and the use of concomitant antidiabetic medications. Physiological variability, including individual incretin responses and glycemia-related hormonal fluctuations, may also contribute. A similar pattern was observed in BMI, which declined from a median of 33.6 kg/m² (IQR 31.0–37.0) to 30.9 kg/m² (IQR 28.7–34.0), with the mean BMI decreasing from 34.8 to 32.0 kg/m² (p<0.001), approaching the threshold for Obesity Class 1. This underscores the effectiveness of semaglutide in addressing weight-related metabolic risk. Glycemic control improved in parallel, with median HbA1c levels declining from 7.80% to 6.90% and mean values from 8.05% to 6.89% (p<0.001), nearing the recommended target of <7% for patients with type 2 diabetes A total HbA1c reduction of 1.9% was observed over the one-year period, highlighting the robust glucose-lowering efficacy of semaglutide, as emphasized in the 2022 ADA/EASD consensus report. Notably, the UK Prospective Diabetes Study (13) demonstrated that each 1% decrease in HbA1c is associated with a substantial reduction in the risk of microvascular complications in patients with T2DM, which is particularly relevant given the high prevalence of such complications in our study population. This improvement aligns with the recommended HbA1c target of <7%, which is suggested by the 2023 European Society of Cardiology (ESC) Guidelines for the management of cardio-vascular disease in patients with diabetes to reduce the risk of microvascular complications (14). These findings have several implications for clinical practice, particularly in the context of the Bulgarian healthcare system. The study cohort included a near-equitable distribution of male and female participants, providing a balanced demographic context for the interpretation of treatment outcomes. In Bulgaria, access to GLP - 1 receptor agonists such as semaglutide is regulated by the NHIF, which requires therapy initiation and ongoing evaluation by a specialist endocrinologist. This framework, while administratively complex, ensures regular specialist follow-up, timely assessment of treatment efficacy, and support for adherence: factors that may contribute to the favorable outcomes observed in this cohort. Despite the procedural requirements for prescribing and renewing therapy, patient access to semaglutide is not significantly hindered due to the widespread availability of endocrinology specialists. The structured monitoring process may partially explain the high proportion of treatment responders and the absence of BMI deterioration in any subgroup. These real-world outcomes confirm that, under appropriate specialist supervision, the clinical benefits of semaglutide observed in randomized controlled trials can be effectively replicated in routine practice, including improvements in both glycemic control and weight management. Our study has certain limitations. Beyond baseline BMI, the response to semaglutide therapy is likely influenced by a range of clinical and behavioral factors that were not accounted for in this analysis. Although all participants received standardized dietary and physical activity counseling as part of the institutional diabetes care protocol, individual adherence to these recommendations was not monitored or quantified. Other potentially influential factors such as the degree of insulin resistance, dietary habits, levels of physical activity, the presence of comorbid conditions (e.g., cardiovascular or renal disease), concomitant pharmacotherapy, and broader social determinants of health, were also not systematically evaluated. Additional variability may result from intercurrent illnesses and differences in adherence to treatment. Although these factors are well recognized in clinical practice, they were not systematically evaluated in the present study and should be considered in the design of future prospective investigations. Another important limitation is the absence of a control group receiving standard clinical treatment, which would permit a more rigorous comparative assessment and adjustment for potential confounders. As this was a retrospective real-world analysis of patients under structured NHIF-mandated dispensary monitoring who had already initiated semaglutide, the inclusion of such a comparator group was not feasible within the available dataset. Nevertheless, these limitations do not undermine the overall validity of the findings. Prospective, controlled studies with longer follow-up are warranted to confirm these results, isolate the independent effects of semaglutide, and evaluate the sustainability of the observed metabolic benefits as well as their impact on diabetes-related complications.

## 5 Conclusions

The initiation of subcutaneous semaglutide in patients with T2DM demonstrates significant benefits in addressing metabolic abnormalities, including hyperglycemia and overweight, within real-world clinical settings. Semaglutide treatment was associated with clinically meaningful and significant improvements in HbA1c, BMI, and body weight from baseline. These findings underscore the efficacy of semaglutide as a therapeutic option in routine clinical practice for the long-term management of T2DM in Bulgaria. Furthermore, the structured monitoring and prescription requirements mandated by the Bulgarian National Health Insurance Fund (NHIF), which involve regular follow-up by endocrinology specialists, may play a supportive role in enhancing patient adherence and achieving favorable outcomes. Despite the administrative complexity of this process, access to care remains timely due to the widespread availability of specialists. To further validate these findings, future research should explore the long-term sustainability of treatment effects beyond one year and assess additional clinical and behavioral predictors of response. Prospective, controlled studies will be crucial to optimize patient selection and improve therapeutic outcomes.

## Data Availability

The datasets presented in this study can be found in online repositories. The names of the repository/repositories and accession number(s) can be found in the article/supplementary material.
